# Hosts winnow symbionts with multiple layers of absolute and conditional discrimination mechanisms

**DOI:** 10.1098/rspb.2022.2153

**Published:** 2023-01-11

**Authors:** Angeliqua P. Montoya, Camille E. Wendlandt, Alex B. Benedict, Miles Roberts, Jonah Piovia-Scott, Joel S. Griffitts, Stephanie S. Porter

**Affiliations:** ^1^ School of Biological Sciences, Washington State University, Vancouver, WA 98686, USA; ^2^ Department of Microbiology and Molecular Biology, Brigham Young University, Provo, UT 84602, USA

**Keywords:** symbiosis, host discrimination, partner choice, indirect genetic effects, legume, rhizobium

## Abstract

In mutualism, hosts select symbionts via partner choice and preferentially direct more resources to symbionts that provide greater benefits via sanctions. At the initiation of symbiosis, prior to resource exchange, it is not known how the presence of multiple symbiont options (i.e. the symbiont social environment) impacts partner choice outcomes. Furthermore, little research addresses whether hosts primarily discriminate among symbionts via sanctions, partner choice or a combination. We inoculated the legume*, Acmispon wrangelianus,* with 28 pairs of fluorescently labelled *Mesorhizobium* strains that vary continuously in quality as nitrogen-fixing symbionts. We find that hosts exert robust partner choice, which enhances their fitness. This partner choice is conditional such that a strain's success in initiating nodules is impacted by other strains in the social environment. This social genetic effect is as important as a strain's own genotype in determining nodulation and has both transitive (consistent) and intransitive (idiosyncratic) effects on the probability that a symbiont will form a nodule. Furthermore, both absolute and conditional partner choice act in concert with sanctions, among and within nodules. Thus, multiple forms of host discrimination act as a series of sieves that optimize host benefits and select for costly symbiont cooperation in mixed symbiont populations.

## 1. Introduction

In durable mutualisms, hosts discriminate among symbionts that differ in the benefits they confer, to preferentially interact with the most beneficial symbionts. It is thought that by allocating greater resources to more beneficial symbionts, hosts optimize their fitness as well as select for symbionts that invest in benefits to hosts. However, we have a limited understanding of the qualities that determine which symbionts are ultimately successful on a host, especially for co-evolving partners from wild populations. Host discrimination can occur in advance of resource exchange via partner choice whereby hosts gain a fitness advantage from preferentially associating with symbionts that confer greater benefits [1,2]. Host discrimination can also occur via sanctions, whereby hosts assess the benefits or costs of exchange with different symbionts they associate with and confer more resources to more beneficial symbionts [3,4]. There is evidence for robust partner choice and sanctions across diverse mutualisms, but most studies identify only one of the two mechanisms at work [4–9]. Despite the importance of partner choice and sanctions for the maintenance of mutualism, relatively little is known about how variation in the community of potential partners (i.e. the symbiont social environment) influences partner choice, how partner choice influences host fitness and whether hosts simultaneously employ both partner choice and sanctions.

In many-to-one mutualisms where multiple symbionts interact with a single host, the extent to which one symbiont influences the resources other symbionts acquire from the host are largely unexplored [9–11]. However, recent research reveals that the resources a symbiont receives in mutualism are conditional: in the legume–rhizobium mutualism plants exert conditional sanctions by conferring greater resources to a given rhizobial symbiont if its nitrogen fixation is superior to that of other available symbionts, but lesser resources if its nitrogen fixation is inferior to that of other available symbionts [9]. Thus, the composition of the symbiont social environment impacts partner sanctions as plants measure and respond to the benefits multiple symbionts confer. However, less is known about the impacts of the symbiont social environment on partner choice.

Under *absolute partner choice*, the initiation of symbiosis by a given symbiont depends solely on its own attributes—a direct genetic effect (DGE). In this scenario, we could predict which symbionts will succeed under simple coevolutionary dynamics for host–symbiont compatibility without influence from the social environment. By contrast, under *conditional partner choice*, initiation of symbiosis with a given symbiont depends on attributes of symbiont genotypes present in the social environment (i.e. genes residing in other symbionts that have the potential to colonize the host)—a social genetic effect (SGE, or indirect genetic effect, IGE) [12–14]. This would generate complex coevolutionary dynamics in models of the evolution of cooperation, such that the form and magnitude of frequency-dependent dynamics due to SGEs could dominate mutualism evolution.

There are distinct ways in which the symbiont social environment could impact mutualism outcomes, with divergent evolutionary implications. Under ‘main SGEs’ a given symbiont genotype in the social environment has a consistent effect on symbiosis outcomes across all conspecific genotypes, such as when the presence of one highly competitive symbiont genotype reduces the rate of colonization by all other symbiont genotypes by a consistent amount. Main SGEs interact with DGEs in an additive manner (G + G) which results in transitive dynamics [13,14]. Transitive dynamics generate a single-best symbiont fitness hierarchy theoretically predicted to purge genetic variation [13,15] from symbiont populations. By contrast, under ‘G × G SGEs’, a given symbiont could have idiosyncratic effects on symbiosis outcomes for different symbiont genotypes in the social environment [14] whereby a symbiont genotype may be highly competitive against one symbiont genotype but not another. G × G SGEs result in intransitive dynamics that can lead to an idiosyncratic symbiont fitness hierarchy predicted to maintain genetic diversity [15]. Therefore, intransitive dynamics among symbionts could be one mechanism that contributes to the maintenance of partner quality variation and consequently host discrimination [16,17].

Although theory predicts that partner choice will benefit hosts [18], little empirical evidence supports this assumption. Simonsen & Stinchcombe [19] find that hosts that preferentially associate with a superior symbiont are favoured by natural selection; however, this study examines selection on hosts exposed to only one effective and one ineffective symbiont genotype, so it is unclear how broadly applicable these results will be across diverse symbiont populations. The adaptive value of partner choice to a host may hinge on its effectiveness in natural populations or the host's ability to use sanctions [1,2,20]. If partner choice does not benefit the host, this could indicate that partner choice is costly or ultimately unsuccessful at constraining the consumption of resources by less-beneficial symbionts [1,2,21].

Empirical studies of host discrimination often test for evidence of either partner choice or sanctions, so we lack a coherent picture of whether multiple layers of host discrimination can act in concert [22–26]. On one hand, effective partner choice could relax selection on hosts to impose sanctions (or vice versa), such that only one form of host discrimination is active in a mutualism, which we term the relaxed selection hypothesis for partner discrimination. On the other hand, imperfect host discrimination mechanisms could favour the maintenance of multiple levels of host discrimination as a series of sieves to winnow the symbiont population and select for optimal partners, which we term the multiple sieves hypothesis. Hosts often compartmentalize symbionts in specialized physical modules such as fruits, organs or root nodules, and assess symbiont quality and confer resources to the best-performing symbiont modules [4,6,27,28]. Sanctions at the level of host modules have been presented as a robust form of host discrimination that maintains cooperation [3,8,9,26,29,30]. For example, fig trees abort fruits when too many fig-wasp eggs are present [4,6] and legume hosts can cut off oxygen [3] or allocate less resources [7,8] to nodules that contain rhizobium that do not fix nitrogen. However, partner choice is also present in many mutualisms and can constrain the particular symbionts able to colonize host modules. While the mechanisms that underlie partner choice are often unknown, colonization of hosts by symbionts is a joint phenotype that depends on the host genotype, symbiont genotype and their interactions [2,31]. Under partner choice, symbiont occupancy patterns on a host could be shaped directly by host control and/or by interactions among symbionts, which are not presently feasible to distinguish. Partner choice is often ineffective if hosts are presented with near-isogenic mutants or other symbiont genotypes with which they lack a coevolutionary history [2,8,9,32,33].

We investigate how continuous natural variation in symbiont genotypes impact host discrimination in the symbiosis between legumes and rhizobia. Leguminous plants house rhizobium bacteria in root nodules, which are commonly infected by single strains but may be infected by multiple strains [34]. Here, rhizobia convert nitrogen from the air into a form useable for plants, essentially fertilizing the plant, in exchange for carbon the plant derives from photosynthesis [28]. We transformed eight *Mesorhizobium* strains to express fluorescent markers. These strains ranged continuously from unbeneficial to highly beneficial [35] and were isolated from wild-collected nodules [36]. We inoculated all 28 factorial pairs of these marked strains onto their native legume host, *Acmispon wrangelianus*. Fluorescent markers allowed us to measure symbiont fitness for co-infecting strains on a plant simultaneously. To understand how symbiont genetics impact partner choice outcomes, we use a quantitative genetic framework to ask, (i) are patterns of symbiont and host fitness consistent with partner choice and is partner choice conditional or absolute? (ii) Are symbiont SGEs transitive or intransitive? (iii) Does partner choice optimize host fitness? (iv) Does partner choice act in concert with other layers of host discrimination?

## 2. Methods

### (a) Bacterial transformations with fluorescent constructs

**Figure 1.**
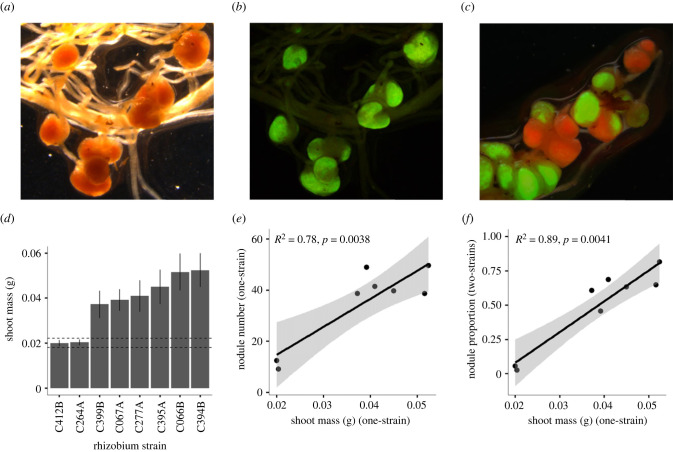
Evidence for partner choice: more beneficial symbionts form more nodules in one- and two-strain inocula. Nodules occupied by a *mEGFP-*labelled *Mesorhizobium* strain in a (*a*) darkfield image and (*b*) with Blue LP filter. (*c*) Darkfield, Blue LP and TXR overlay image of nodules containing strains marked with *mEGFP* and *mScarlet-l*. (*d*) Shoot mass of *Acmispon wrangelianus* SM2A9 in symbiosis with a one-strain inoculation of each *Mesorhizobium* strain. Bars, mean effects +/− s.e.; dotted lines, +/− one s.e. for plant shoot mass in the absence of any rhizobia. More beneficial *Mesorhizobium* strains form more nodules in (*e*) one-strain inoculations and in (*f*) two-strain inoculations of *A. wrangelianus*. Filled circles, genotype mean values for each *Mesorhizobium* strain. Black line, linear regression. Grey shading, 95% confidence interval.

We investigated eight *Mesorhizobium* strains isolated from wild-collected nodules of *Acmispon wrangelianus* [36]. To visually differentiate between strains in experimental nodules, we transformed each strain to produce one red and one green fluorescent isogenic descendent. To do so, we transformed strains with plasmid constructs containing a neomycin antibiotic resistance gene and either monomeric-enhanced green fluorescent protein (*mEGFP*) [37] (figure 1*a,b*) or monomeric red fluorescent protein (*mScarlet-l*) [38] (figure 1*c*). The plasmids (electronic supplementary material, figure S1) were first introduced into specialized *E. coli* donor strain, MFDpir [39], using bacterial transformation. MFDpir donor and *Mesorhizobium* recipients were mixed as bi-parental matings [40], to mobilize the fluorescent protein expression vectors into the eight recipient *Mesorhizobium* strains (details in electronic supplementary material). Fluorescence expression was visualized on a Leica M165 FC Fluorescent Stereo Microscope using filters ‘TXR’ (excitation 560 nm, emission 610 nm long pass) for *mScarlet-l* [38] or ‘Blue LP’ (excitation 470 nm, emission 515 nm long pass) for *mEGFP* [37].

### (b) Greenhouse experimental design

We grew *Mesorhizobium* in symbiosis with a single genotype of the native *Acmispon wrangelianus* host plant as both one-strain inoculations (eight treatments) and all possible two-strain combinations (28 treatments), replicated over eight blocks. To control for possible differences in the effects of the two fluorescent markers, each strain was labelled separately with each of the two markers. We used reciprocally marked two-strain inocula within each block (i.e. each two-strain combination was grown twice, once with each possible combination of the two markers). Each block also had two uninoculated control plants, for a total of 592 plants in a randomized complete block design in the Washington State University Vancouver greenhouse (45.7328054° N, 122.635967° W).

### (c) Plant cultivation and inocula

*Acmispon wrangelianus* genotype SM2A9 seeds were planted on 14 February 2020 and cultivated following Porter *et al*. [35] (details in electronic supplemental material). Seeds germinated and established for 6 weeks in a greenhouse with 14 h, 21°C days and 10 h, 18°C nights [35]. To water the plants without releasing transgenic *Mesorhizobium*, plant pots were placed in 175 ml test tubes containing 20 ml of sterile water, which was replenished throughout the experiment. *Mesorhizobium* strains were grown on TY agar [41] with neomycin for 72 h at 28°C, rinsed and resuspended at 6 × 10^6^ cells per 900 µl (based on OD_600_) in sterile water on ice. Two-strain inocula were prepared by combining equal volumes of each strain. Each host plant was inoculated with 900 µl of inoculum or a sterile water control by pipetting onto the soil at the base of the plant. Plants were fertilized every other week (three times total) (details in electronic supplementary material). Plants were harvested one block per day starting 8 May 2020. Roots were washed and stored at 4°C. Shoots were clipped, dried to constant temperature at 60°C and weighed.

### (d) Symbiosis phenotyping

Fluorescent root nodules were counted on a fluorescence stereomicroscope within two weeks of harvest. We inferred rhizobium fitness as the number and proportion of nodules formed by a strain. We assigned each strain a count of 1 for a single-colour nodule and a count of 0.5 for a mixed-colour nodule. We assessed within nodule fitness for rhizobia in two experimental blocks. We inferred rhizobium fitness within a nodule as the number and proportion of rhizobium colony-forming units (CFUs) per nodule. We extracted nodules at random from each plant to determine the number and colour of CFUs per nodule within a day of harvest, following Wendlandt *et al*. [42] (details in the electronic supplementary material). We extracted one nodule from each plant receiving one-strain inocula. We extracted one mixed-colour nodule and two single-colour nodules, when possible, from each plant receiving two-strain inocula.

### (e) Statistical analyses

#### (i) Partner choice and sanctions

To test for partner choice, we examined whether strains that confer higher plant shoot mass in one-strain inocula: (i) form more nodules in one-strain inocula and (ii) form a higher proportion of nodules in two-strain inocula. To test for sanctions within nodules for plants that received two-strain inocula, we tested whether more beneficial strains: (i) form a greater number of CFUs in nodules containing a single strain (among nodule sanctions) and (ii) form a greater proportion of CFUs in nodules containing two-strains (intra-nodule sanctions). Models used fixed effects linear regression on strain genotypic means.

#### (ii) Absolute and conditional partner choice components

We quantified the impact of the rhizobium social environment—both additive (DGE and main SGE) and non-additive (G × G SGEs) genetic effects—on plant and rhizobium symbiotic fitness. For plants receiving two-strain inocula, we used shoot mass as a proxy for this annual plant's fitness, and the number and proportion of nodules founded by a strain on a plant as proxies for rhizobium fitness (details in the electronic supplementary material and figure S2). Absolute partner choice occurs if more beneficial strains initiate more nodules on a plant due to the impact of this focal strain's own genes on its symbiotic fitness (DGE). Conditional partner choice occurs if nodulation by a focal strain is impacted by the genes in a competitor strain. This includes the extent to which competitor rhizobia exert a consistent effect on nodulation by focal strains (main SGE) and the extent to which competitor rhizobia exert a variable effect on nodulation by focal strains (G × G SGE).

We estimated the proportion of total phenotypic variance in plant and rhizobium fitness explained by the genotype of a focal rhizobium strain (DGE), the genotype of a competitor strain (main SGE), their interaction (G × G SGE) and block, as random effects in linear mixed models in ASReml-R v4.1 [43] with Gaussian residuals, to generate parameter estimates that are readily interpretable [13]. Fluorescent marker was modelled as a fixed effect. For the proportion of nodules formed by a focal strain and plant shoot mass, strain designation as focal or competitor was unbiased so the genotypic effect of a strain does not depend on the strain's designation as focal or competitor. Therefore, for these phenotypes, we constrained the variance in mixed effects models so that DGE = main SGE for each strain, following Lane *et al*. [13]. The within-individual correlation between focal and competitor was set to 1 for the shoot mass model, and −1 for nodule proportion since the proportion of nodules filled by each of the two-strains on a plant is perfectly negatively correlated. For nodule number, no variance constraints were used and plant pot was included as a random effect due to non-independence of the two observations per pot. Following a similar analysis by Lane *et al*. [13], we used a Gaussian distribution. Other distributions in ASReml use penalized quasi-likelihood, which can perform poorly in estimating variance components [43]. Fit of the data for all response variables to the distributional assumptions of the models was checked using diagnostic plots. A log transform improved the fit of shoot mass to the assumptions of the Gaussian distribution and these data are presented. Nodule proportion data are largely consistent with the assumptions of a Gaussian model as the distribution of residuals is unimodal, symmetrical, and not heavily skewed towards the boundaries, and was left untransformed in analysis. A log transform did not alter patterns of significance for nodule number and findings for untransformed nodule number are presented. We used likelihood ratio tests to determine significance of the random effects. Where DGE and main SGE were constrained to be equal, we removed both the model terms and their correlation structure to simultaneously test DGE and main SGE.

#### (iii) Benefits of partner choice

To determine if plants benefit from exercising stronger partner choice, we modelled how plant fitness deviates from the neutral expectation in the absence of partner choice. In the absence of partner choice, we expect two strains inoculated in equal ratios should form equal numbers of nodules and host fitness should then be the average of the host's fitness in one-strain inocula with these strains [8,44,45]. We scaled this deviation to the size of the mean host fitness to indicate proportional changes as follows:

$$\begin{array}{rl} & \text{Deviation from neutral expectation}\\ & \;=\;\frac{W_{P\_R1,Rs}-((W_{P\_R1}+W_{P\_R2})/2)}{\left((W_{P\_R1}+W_{P\_R2})/2\right)}, \end{array}$$

where *W_P_R1,R2_* indicates host fitness in two-strain inocula, and *W_P_R1_* and *W_P_R2_* indicates host fitness in each of the one-strain inocula. Deviations greater than 0 and positively correlated with partner choice indicate a fitness benefit of partner choice for the host (details in the electronic supplementary material). We modelled the deviation from a neutral expectation for plants that received two-strain inocula using a linear mixed model, with a fixed effect of proportion of nodules initiated by the more beneficial strain, and random effects of strain ID, competitor strain ID, and block. Running this model with a log transformation alleviated heterogeneity of variance in the residuals, but did not qualitatively alter results. All analyses were conducted in R v. 4.0.3 [46].

## 3. Results

### (a) Partner choice

We find robust partner choice in the symbiosis. In one-strain inocula, the eight *Mesorhizobium* strains vary continuously in benefit to the *Acmispon wrangelianus* host (figure 1*d*). For plants inoculated with one-strain inocula, more beneficial strains formed more nodules, even though there was only a single symbiont available to the host (*F*_1,6_ = 20.9, *p* = 0.004; figure 1*e*). This pattern is not due simply to larger plants forming more nodules. Even when we treat nodulation and shoot mass as an allometric trait that accounts for variation in plant size in response to rhizobium quality (nodule number per gram of shoot mass), we find more beneficial strains found more nodules per gram of shoot mass in one-strain inocula (*F*_1,6_ = 7.3, *p* = 0.036; electronic supplementary material, figure S3). Some strains formed few nodules compared to other strains in one-strain inocula, and this could impact the benefits the host obtained from nodules. To account for this variation, we also measure host benefit on a per nodule basis (shoot mass (g) per nodule) and find that more beneficial strains provide greater shoot mass increase per nodule than the strains that form few nodules in one-strain inocula, which provide no benefit per nodule to the host (*F*_1,6_ = 143.1, *p* < 0.001; electronic supplementary material, figure S4). Therefore, the benefits plants obtain in one-strain inocula are not simply driven by the number of nodules formed on the host, but also by differences in the benefit each nodule provides. We also measured each strain's intrinsic axenic growth rate and found no relationship with nodule number in one-strain inocula (*F*_1,6_ = 1.0, *p* = 0.35) or nodule proportion in two-strain inocula (*F*_1,6_ = 2.4, *p* = 0.17). This indicates that strains that nodulate more do not do so due to faster growth rates (details in the electronic supplementary material). For plants inoculated with two-strain inocula, a higher proportion of nodules on a host plant were formed with the more beneficial strain (*F*_1,6_ = 49.4, *p* < 0.01; figure 1*f*). Thus, partner choice is also evident when multiple partners are available to the host.

### (b) Partner choice has both absolute and conditional components

**Figure 2.**
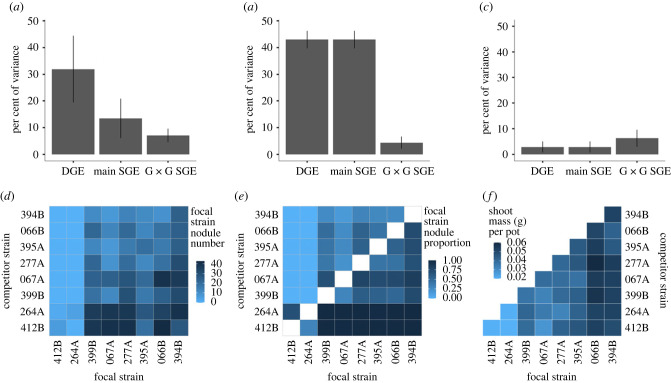
Symbionts that vary in benefit to the host experience absolute and conditional partner choice. DGEs, main SGEs and genotype-by-genotype (G × G SGEs) impact *Mesorhizobium* and *Acmispon wrangelianus* fitness. Shown is the per cent of the phenotypic variance in (*a*) nodule number, (*b*) nodule proportion and (*c*) shoot mass, with complementary heat maps, respectively (*d*–*f*). Heat maps show the (*d*,*e*) focal strains fitness against each competitor or (*f*) host fitness in each one-strain combination. Heat map diagonal in (*d*) is the one-strain inocula nodule number halved to compare with two-strain inocula nodule number, in (*e*) no biological data, and in (*f*) is the host shoot mass in one-strain inocula.

When two strains are present, a strain's ability to nodulate depends on both its own genetics (DGE), as well as the strain genotype present in the social environment, via both main SGEs and G × G SGEs. For nodule number, 31.9% of variation is explained by focal strain genotype (DGE), 13.5% is explained by competitor strain genotype (main SGE), and 7.1% is explained by the G × G interaction of focal and competitor strains (G × G SGE) (table 1; figure 2*a*,*d*). For the proportion of nodules formed by a strain, 86.0% of the variation is explained by the additive effects of DGE and main SGE (table 1; figure 2*b*,*e*). Non-additive G × G SGEs explained 4.4% of the total variation (table 1; figure 2*b*,*e*). We observe main SGEs whereby strains affect symbiosis outcomes of other strains similarly (figure 2*d*; electronic supplementary material, figures S5–S7). We also observe G × G SGEs whereby strains have idiosyncratic impacts on symbiosis outcomes for other strains such that symbiosis outcomes depend on the genotype by genotype interaction between the focal and competing strain (figure 2*d*; electronic supplementary material, figures S5–S7).The rhizobium social environment has a significant but modest effect on host fitness. For plant shoot mass, 6.0% of variation is explained by the combined additive effects of DGE and main SGE, while 6.3% of variation is attributed to the G × G SGE (table 1; figure 2*c*,*f*). It is possible that the large amount variation in *A. wrangelianus* shoot mass that is unexplained by terms in our model results from high plasticity in this aboveground trait in response to variation in light or temperature conditions in the greenhouse that are not accounted for by the block term.

**Table 1.** Both symbiont genotype and the social genetic environment impact partner choice outcomes. Likelihood ratio test *χ*^2^ statistics for random effects in GLMMs modelling rhizobium fitness (nodule number or nodule proportion) and host fitness (shoot mass). DGE, direct genetic effect; SGE, social genetic effect; G × G, genotype by genotype interaction.

component	nodule number			nodule proportion			shoot mass		
	% variation	*χ*^2^	*p*	% variation	*χ*^2^	*p*	% variation	*χ*^2^	*p*
DGE	31.9	49.1	<0.001	43	120.6	<0.001	2.9	5.5	<0.01
main SGE	13.5	25.6	<0.001	43	120.6	<0.001	2.9	5.5	<0.01
G × G SGE	7.1	49.5	<0.001	4.4	69.6	<0.001	6.3	6.1	<0.01
block	2.8	26.3	<0.001	0.3	4	<0.05	10.9	31.9	<0.001
plant pot	7.1	10.7	<0.001						
residual	37.7			9.4			77		

### (c) Partner choice benefits the host

**Figure 3.**
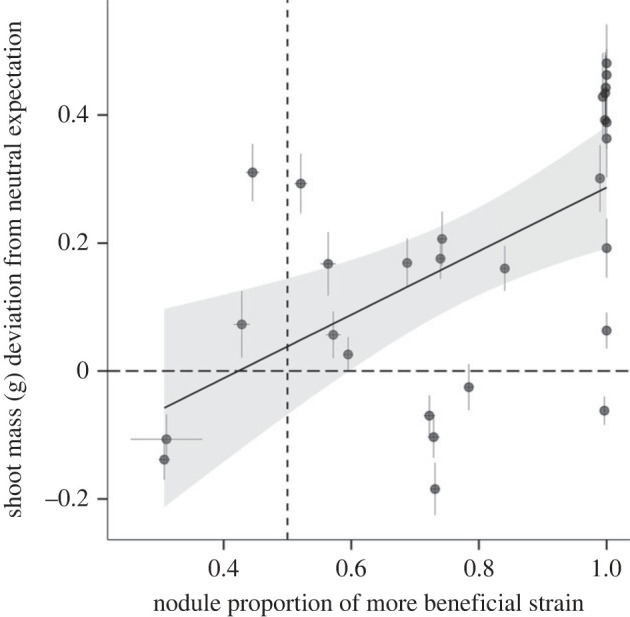
Stronger partner choice yields greater plant fitness across 28 symbiont pairs. Filled circles, mean shoot mass deviation from the expected benefit to the host shoot mass under a neutral expectation (long dashed line) for two-strain inocula. Vertical dashed line, the nodule proportion (0.5) corresponding to no partner choice. Horizontal dashed line, the neutral expectation for host fitness under no partner choice. Black line, linear regression. Grey shading, 95% confidence interval. (Online version in colour.)

We find stronger partner choice allows the host to reap greater mutualism benefits. For two-strain inocula where the inferior strain was more effectively excluded from nodules, the host gains greater fitness benefits from symbiosis, relative to neutral expectation (*χ*^2^ = 10.3, *p* = 0.001; figure 3). For most of the two-strain inocula, partner choice was evident (more than 50% of nodules occupied by the more beneficial strain) and plant fitness exceeded the neutral fitness expectation (figure 3). We find the same result when we calculate the neutral expectation using host fitness on a per nodule basis (*χ*^2^ = 6.9, *p* = 0.009; electronic supplementary material, figure S8). Since the two unbeneficial strains formed few to no nodules in the presence of the more beneficial strains, we acknowledge that the benefit to the host model result may be driven by these strain pairings. However, this pattern whereby unbeneficial strains are excluded from nodules in the presence of a superior strain is exactly what we would expect to observe if partner choice benefits the host.

### (d) Multiple layers of host discrimination: nodule level and intra-nodule sanctions favour beneficial strains

**Figure 4.**
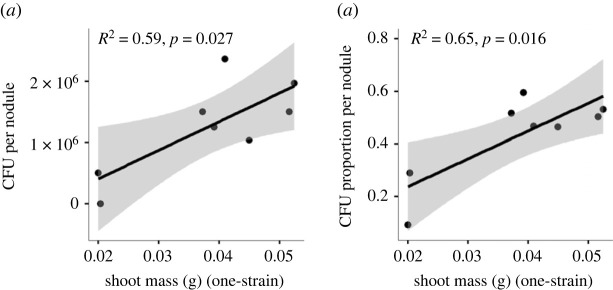
Evidence for sanctions: more beneficial symbionts form more progeny in single strain and co-infected nodules. More beneficial *Mesorhizobium* strains form more CFUs in (*a*) single-strain nodules and a higher proportion of CFUs in (*b*) mixed nodules. Filled circles, genotype mean values for each *Mesorhizobium* strain. Black line, linear regression. Grey shading, 95% confidence interval. (Online version in colour.)

We find that multiple layers of host discrimination act together to favour more beneficial strains. In addition to partner choice, we find evidence of sanctions in two-strain inocula treatments. More beneficial strains obtain higher CFUs per nodule than less-beneficial strains, both for nodules infected by single strains (among-nodule sanctions; *F*_1,6_ = 8.5, *p* = 0.027; figure 4*a*; electronic supplementary material, figure S9*a*) and for nodules infected by two strains (intra-nodule sanctions; *F*_1,6_ = 11.1, *p* = 0.016; figure 4*b*; electronic supplementary material, figure S9*b*).

## 4. Discussion

Understanding how the genetic composition of symbiont communities impacts mutualist partner discrimination is critical to reveal how hosts optimize fitness in the face of diverse symbiont options, and how cooperation in mutualism is maintained despite its costs [1,20,47]. However, little is currently known about how symbiont genetics and the social genetic environment impact partner discrimination. Here, we measure mutualism outcomes for plants exposed to 28 two-strain communities of fluorescently labelled rhizobium strains to uncover how a strain's ability to initiate nodules, and proliferate within them, is affected by its own genetic attributes, as well as those of other strains in the social environment. We find robust partner choice in a native, wild legume–rhizobium symbiosis. Partner choice outcomes are determined not only by a symbiont's own genotype (DGE), but also by SGEs of competitor strains, showing that partner choice has both absolute and conditional components, respectively. In this symbiosis, hosts benefit from more effective partner choice: hosts that more successfully exclude inferior rhizobium strains obtained higher fitness benefits from symbiosis than would be expected in the absence of partner choice. Furthermore, our findings support the multiple sieves hypothesis of host discrimination whereby multiple forms of partner choice and sanctions function to preferentially direct rewards to more beneficial symbionts.

### (a) Absolute and conditional partner choice

We quantify for the first time how variation in symbiont fitness under partner choice is impacted by symbiont SGEs. First, we find robust support for partner choice: while all the wild-collected *Mesorhizobium* strains in this study can nodulate their native host plant, the more beneficial symbionts form more root nodules on plants inoculated with one-strain or two-strain inocula. We also find that symbiont and host fitness under partner choice is impacted by both a symbiont's own genetic attributes (DGEs), an absolute form of partner choice, and by the SGE of competitor genotypes, a conditional form of partner choice. Studies of near-isogenic, non-fixing rhizobium mutants competing against their wild-type ancestor to initiate nodules often observe a lack of partner choice whereby less-beneficial mutants form the same number of nodules as the more beneficial wild-type strain [8,9,32,33]. By contrast, our findings are consistent with studies of natural rhizobium diversity in which hosts are often able to select superior symbionts prior to resource exchange, presumably on the basis of honest symbiont signalling of quality to the host [5,7] or genetic linkage between loci determining symbiont benefit to the host and ability to colonize the host [2].

The additive effects of DGEs and main SGEs impacting a symbiont's ability to colonize a host under partner choice are transitive: a large component of a strain's impact on another strain's ability to form nodules is consistent in its impact across strains in the social environment [13,14]. Theory predicts that these DGEs and main SGEs will tend to result in the loss of genetic variation in the symbiont social environment over many generations [14], leaving only the most competitive strains for nodulation. If host discrimination mechanisms impose generations of positive directional selection on symbiont benefit, the most beneficial symbiont genotype would be expected to increase in frequency, possibly leading to relaxed selection on hosts to maintain discrimination mechanisms [16].

However, we also find that idiosyncratic outcomes between different genotype-by-genotype combinations of symbionts play a role in determining which symbionts are successful in initiating symbiosis. Both the number and proportion of nodules a strain forms are determined in part by G × G SGEs, though the magnitude of this effect is small. This effect is intransitive: a component of a strain's impact on another strain's ability to form nodules is inconsistent in its impact across strains in the social environment [13,14]. This form of intransitive dynamics complicates predictions of which symbionts will ultimately be most successful and thus contributes to the maintenance of symbiont diversity.

Genetic determination of the proportion of nodules that will be formed by one strain or another in a given two-strain *Mesorhizobium* community is remarkably strong. In fact, 86% of the variation in the proportion of nodules founded by a strain is explained by the combined additive effects of a strain's own genotype and the genotype of the competitor strain. A further 5% of the variation in the proportion of nodules founded by a strain is explained by genotype-by-genotype interaction between the co-inoculated strains. Thus only a small amount of variation in this trait is unexplained by genetic sources. This finding is consistent with the lack of environmental sensitivity of nodule occupancy patterns observed in other experiments [7,48]. A strong genetic component to nodule occupancy is also observed when partner choice is lacking: Grillo *et al*. [48] find that even when the most beneficial strain for most plant families changes across contrasting levels of nitrogen fertilization, nodule occupancy remains consistent [5]. The authors hypothesize that the interaction of signalling molecules between hosts and symbionts may work like a ‘lock and key’ system which leads to consistent nodule occupancy across environments [19,44,48].

Symbiont genetic effects on host fitness mirror those for symbiont fitness. The impact a strain has on host fitness is determined both by a strain's own genotype (DGE), as well as the main effect of the competitor strain genotype (main SGE), though the magnitude of these effects is modest. These symbiont genetic effects on host fitness would lead to relatively simple evolutionary trajectories for the symbiosis. However, a symbiont's impact on host fitness is also determined in part by G × G SGEs such that it is necessary to account for idiosyncratic host fitness outcomes for particular strain–strain pairs in order to most accurately model host benefit in any particular symbiont social genetic environment. These findings indicate that models and studies focused on single-symbiont environments will be incomplete in informing how cooperation evolves in one to many mutualisms. We also acknowledge that host legumes likely encounter many more strains in nature than they encounter in our simple two-strain inocula. Furthermore, symbiosis outcomes could depend upon both host and symbiont genotypes, such that symbiont SGEs may also be impacted by variation in host genotype [44]. While we use only a single host genotype to investigate symbiont SGEs, future studies that examine symbiont SGEs across multiple host genotypes could determine whether symbiont SGEs work in tandem with host–symbiont G × G interactions to maintain variation in partner quality.

### (b) Hosts benefit from more effective partner choice

Understanding how costly cooperation traits are maintained in symbionts has long motivated study of host discrimination as a mechanism to select for symbiont cooperation [1,19,49]. To be maintained by natural selection, however, discrimination must provide a fitness benefit to the host. However, while host discrimination to preferentially associate with more beneficial symbionts is demonstrated in many studies [5,7,22–24,50–53], fitness benefit to the host has rarely been tested empirically. We show that not only do hosts benefit from partner choice in the face of diverse symbiont populations, but that host fitness is optimized by stronger partner choice. While hosts can benefit from partner choice when exposed to only one effective and one ineffective symbiont genotype [19], we know of no other empirical evidence that partner choice benefits a host exposed to populations of symbionts that show natural continuous variation in quality. Our deviation from a neutral expectation of host fitness in the absence of partner choice was calculated similarly to Heath & Tiffin's [44] relative performance of host plants grown in two-strain inocula. While they find that some plant populations make more leaves or fruits when inoculated with two-strains of differing quality than expected, they do not measure whether the more beneficial rhizobium had a greater nodule occupancy in co-inoculations and thus do not test whether partner choice benefits the host plant. Using fluorescent markers allows us to show that host plants reap greater fitness benefits when they more effectively initiate nodules with the more beneficial symbiont genotype.

The mechanisms underlying partner choice are still poorly understood. While the molecular mechanisms that determine whether a rhizobium strain can form a nodule on a host genotype are well-studied [54,55], much less is known about mechanisms by which one-strain forms more nodules than another when hosts are presented with mixed symbiont populations. On one hand, partner choice could result from direct host control. Hosts exposed to multiple strains could sense and respond to initial molecular signals, such as nod factors or extracellular polysaccharides that symbionts produce, and preferentially initiate nodules with those symbionts that secrete signals that are linked to higher benefit in symbiosis [55,56]. Generations of coevolution between hosts and symbionts could create genetic linkage between rhizobial loci encoding molecular signals, such as Nod genes, and loci determining symbiont benefit to the host, such as Nif genes that encode the nitrogenase enzyme, many of which are situated in close proximity in the symbiosis island in *Mesorhizobium* [2,57]. On the other hand, partner choice could be an outcome of rhizobium competition. For example, asymmetric competitive advantages [15] in rhizobium competition for nodulation could be driven by the production of lipopolysaccharides, bacteriocins, catabolic proteins for host-derived compounds [2] and differences in strain motility [23]. Here, a hierarchy in nodulation ability could arise if the host creates an environment wherein more beneficial symbionts outcompete less-beneficial symbionts for host colonization similar to screening mechanisms of partner choice in *vibrio–squid* and *ant–acacia* mutualisms [4,27,58,59]. Screening would require genetic linkage between rhizobium alleles conferring high competitive ability for nodulation and alleles conferring high benefit to the host.

### (c) Multiple sieves impose host discrimination

In the *Acmispon wrangelianus*–*Mesorhizobium* mutualism, we find robust partner choice and sanctions at multiple levels of spatial organization on the host, providing support for the multiple sieves hypothesis of partner discrimination. Not only did more beneficial strains initiate more nodules in one- and two-strain inocula (module initiation), more beneficial strains had more progeny within nodules occupied by a single strain (among-module) and within nodules occupied by two strains (intra-module) under sanctions (*sensu* [4]). Symbiosis modules containing more than one symbiont are common in nature [4,6,34,60,61], yet few studies examine partner discrimination across both mixed and single-symbiont genotype populations in modules on a single host. These findings are congruent with those from studies on *Acmispon strigosus* and natural *Bradyrhizobium* symbionts that find sanctions occur both among nodules harbouring different strains and within nodules that contain multiple strains [7,25,62,63]. In nodules, rhizobia cells are enclosed in symbiosome structures and the *Acmispon* host can sanction individual cells at the level of the symbiosome [25]. Refining our understanding of which host discrimination mechanisms are present and the levels at which they act in natural populations of hosts and symbionts may help elucidate how host discrimination evolves [1], and whether mutualisms are stable in the face of cheaters at different levels of symbiont compartmentalization [2,4,17,62,64].

## 5. Conclusion

We reveal that host discrimination has both absolute and conditional components such that both a focal strain's genotype (DGEs) and the genotype of other co-infecting strains (SGEs) modulate the amount of mutualism resources a host confers to a symbiont. We expect that as the symbiont social environment evolves, this will alter fitness landscapes for symbionts and the hosts they colonize. Future work that examines the genetic basis for competitiveness for host colonization may help resolve mechanisms of partner choice: to what extent are more beneficial strains successful due to active choice by the host or due to screening [27] whereby the symbionts that are most beneficial to the host are also those that are most competitive? Our findings highlight that to understand the evolution of host–symbiont mutualisms, we not only need to identify the molecular variants that mediate loci and molecules by which partners interact [60,65,66], we also need to understand the distribution of quantitative variation for cooperation traits in natural populations.

## Data Availability

Data and code are available in the Dryad Digital Repository: https://doi.org/10.5061/dryad.bk3j9kdgd [67]. The supplementary data are provided in the electronic supplementary material [68].
